# Clinical outcomes of solid organ transplant patients after total joint arthroplasty: a propensity-matched analysis

**DOI:** 10.1007/s00590-026-04740-y

**Published:** 2026-06-01

**Authors:** Farouk Khury, Braden V. Saba, Jean Shanaa, Joshua C. Rozell, Vinay K. Aggarwal, Ran Schwarzkopf

**Affiliations:** 1 Technion Israel Institute of Technology, The Ruth and Bruce Rappaport Faculty of Medicine, Technion City, Haifa, 3200003 Israel; 2 Department of Orthopedic Surgery, Rambam Health Care Campus, Haifa, Israel; 3 Department of Orthopedic Surgery, NYU Langone Health, New York, NY USA

**Keywords:** Solid organ transplant arthroplasty, SOT TJA, SOT THA, SOT TKA, Solid organ transplant joint replacement

## Abstract

**Background:**

Solid organ transplant (SOT) patients undergoing total joint arthroplasty (TJA) may be at higher risk for complications due to complex medical and surgical histories, chronic immunosuppressive medications, and significant ongoing comorbidities. This study aimed to evaluate postoperative outcomes following primary, elective TJA in patients with a history of SOT.

**Methods:**

We retrospectively reviewed 53,043 primary, elective TJA patients from 2011 to 2025. Patients were screened for SOT history prior to TJA. All SOT patients were taking some form of immunosuppressive medication following their transplantation. Demographics, SOT details, and surgical data were obtained. SOT patients (*n* = 70) underwent a nearest-neighbor 1:3 propensity-score matching to non-SOT (NSOT) controls (*n* = 210) based on age, sex, smoking, Charlson Comorbidity Index, body-mass index, and TJA indication. Kidney transplants were most common (61.4%), followed by liver (24.3%), and heart (8.6%). Differences in surgical outcomes and postoperative complications between the patients were investigated using *Chi*-squared tests, independent *t*-tests and effect size (ES) estimates. Baseline characteristics did not differ between the groups (*P* > 0.05).

**Results:**

SOT patients had significantly longer hospital stays (92 vs. 51 h, *P* < 0.001, *ES* = 0.82), higher rates of discharge to skilled nursing facilities (SNF) (15.7% vs. 5.7%, *P* = 0.014, *ES* = 0.17) and all-cause 90 day readmissions (15.7% vs. 6.7%, *P* = 0.040, *ES* = 0.12), primarily driven by non-surgical reasons (14.3% vs. 4.3%, *P* = 0.010, *ES* = 0.16) compared to NSOT patients. All-cause revision rates were comparable between SOT and NSOT patients (4.3% vs. 3.8%, *P* = 0.999), including aseptic (2.9% vs. 1.9%, *P* = 0.642) and septic causes (1.4% vs. 1.9%, *P* = 0.999).

**Conclusions:**

Despite higher rates of SNF discharge and non-surgical 90 day readmissions, SOT patients achieved similar all-cause, septic, and aseptic revision rates, compared to NSOT patients. These findings suggest that compared to well-matched comorbid controls, SOT patients can safely undergo elective TJA with comparable revision risk. Enhanced perioperative care may help reduce readmission risks in this complex population.

## Introduction

Total joint arthroplasty (TJA) is widely considered one of the most successful and cost-effective treatments for end-stage hip and knee disease, offering substantial improvements in pain, mobility, and quality of life [1, 2]. Advances in surgical techniques, perioperative care, and implant design have broadened the indications for total hip and knee arthroplasties (THA and TKA, respectively), making it accessible to increasingly complex patient populations [3–6]. Among these are solid organ transplant (SOT) recipients, whose life expectancy has improved considerably over the past several decades due to advancements in transplant surgery, immunosuppressive therapy, and long-term graft management [7, 8]. Thus, the population of SOT recipients undergoing TJA is growing as transplant survival improves, along with age-related joint degeneration

Patients with a history of SOT present unique challenges in the context of elective arthroplasty. Chronic immunosuppression, while essential for graft survival, can impair wound healing, increase susceptibility to infection, and exacerbate underlying comorbidities [9–11]. Many SOT recipients have significant medical and surgical histories, reduced physiologic reserve, and increased frailty, all of which may further elevate their perioperative risk [12–14]. As a result, their outcomes following TJA may differ from those of the general population, even when matched for baseline comorbidities.

Existing literature on arthroplasty outcomes in SOT recipients is limited and mostly consists of small, single-institution retrospective series [15]. While some studies suggest increased complication rates and resource utilization in this population, few have leveraged propensity-score matching to isolate the effect of transplant history from that of overall medical complexity, limiting the ability to determine whether worse outcomes in this population are due to transplant history itself or to associated comorbid conditions [16, 17]. Furthermore, the drivers of adverse postoperative outcomes, particularly whether complications are predominantly surgical or medical in nature, remain poorly defined. Understanding the source of these unfavorable events is critical for optimizing perioperative management and guiding surgical decision-making in the medically complex SOT TJA population.

The purpose of this propensity-score matched study was to compare perioperative outcomes, 90 day readmission rates, and revision risk following primary, elective TJA in SOT recipients vs. non-SOT (NSOT) controls matched for key demographic and comorbidity variables. We hypothesized that SOT recipients would have higher rates of adverse events, even after matching, with differences primarily driven by non‑surgical complications.

## Methods

### Study design

A retrospective study was conducted at an urban, academic institution identifying an initial pool of 53,043 patients who underwent primary THA or TKA between June 2011 and March 2025. This cohort was subsequently screened to investigate patients who underwent their arthroplasty following a previous SOT (heart, kidney, liver, lung, or pancreas). Following approval by the Institutional Review Board (IRB), a retrospective review was conducted of patient records and corresponding data to identify patients who underwent primary THA or TKA indicated for primary or secondary osteoarthritis, avascular necrosis of the femoral head, or femoral neck fracture, and had complete electronic records with at least 90 days of follow-up visits detailing their postoperative progress as well as development of any complications, readmissions, reoperations or revisions. Patients were excluded from the study if they underwent unicompartmental knee arthroplasty, hemiarthroplasty, bilateral arthroplasty, revision or conversion arthroplasty, if they had any diagnosis of ongoing or previous soft tissue, bone, or joint infection, or if they had any missing data. Initial query identified 98 potential patients. The charts of these 98 patients were reviewed individually. Following exclusion of 24 patients who had less than 90 days of follow-up and four patients with missing data, the SOT cohort consisted of 70 patients who underwent THA (*n* = 36) and TKA (*n* = 34) following SOT. The final 70 SOT patients were propensity-score matched to a control group of patients in a 1:3 nearest-neighbor fashion.

### Data collection and outcome measures

Our institution’s electronic medical records (EMR) system (Epic Caboodle. Version 15; Verona, Wisconsin, USA) was utilized to collect baseline demographics, including age, sex, race, smoking status, American Society of Anesthesiologists (ASA) score, body mass index (BMI), and Charlson Comorbidity Index (CCI), discharge disposition, length of stay (LOS), duration of surgery, 90 day readmissions, reoperations and revisions. Propensity-score matching (PSM) was based on age, sex, BMI, race, smoking status, CCI, and procedure indication (osteoarthritis, avascular necrosis of the femoral head, or femoral neck fractures). All patients included in the study were maintained on immunosuppressive therapy following their SOT. PSM was conducted using these demographic variables to create a similar cohort of THA and TKA recipients without SOT, noted as NSOT. The propensity scores were estimated using logistic regression, and matching was performed using the Nearest Neighbor method. Following matching, the standardized mean differences for all covariates were below 0.25 (age: 0.122, sex: 0.175, BMI: 0.103, race: 0.062, smoking status: 0.077, CCI: 0.031, and procedure indication: 0.128) – indicating adequate balance between both patient groups. After matching the cohorts, *chi*-squared (x^2^) tests and independent samples *t*-tests confirmed that there were no significant demographic differences between the SOT and NSOT patients.

### Data analyses

Baseline patient characteristics were represented as counts with percentages for categorical variables and means with standard deviations for continuous variables. In addition to descriptive statistics, the model consisted of *Chi*-squared (x^2^) tests for assessing the differences in terms of categorical outcomes (sex, race, smoking status, ASA score, procedure, procedure indication, discharge disposition, 90 day readmissions (both surgical and non-surgical), aseptic and septic revisions), and independent sample t-test to evaluate the differences of the means of continuous variables (age, BMI, CCI, LOS, and duration of surgery) between the SOT and NSOT patients. Significance was defined as a *P*-value less than 0.05. In cases where a significant finding was observed, effect size (ES) was calculated for Cohen’s *d* (numerous parameters: 0.2–0.5 small, 0.5–0.8 medium, > 0.8 large) and Cramer’s *V* (categorical parameters: 0.1–0.3 small, 0.3–0.5 medium, > 0.5 large). Data was organized and stored using Microsoft Excel software (Microsoft Corporation, Richmond, WA). PSM was performed using R software (Version 4.4.0; R Foundation for Statistical Computing, Vienna, Austria). *Chi*-squared (x^2^) tests and independent samples *t*-tests were conducted using SPSS Statistics (Version 28; IBM Corporation, Armonk, New York, USA).

### Baseline demographics

A total of 280 patients (70 SOT and 210 NSOT) were included in the study. Table 1 lists the differences in SOT and NSOT patient demographics. Mean age at surgery was 63.3 years (range, 34–83) for the SOT patients and 64.2 years (range, 24–91) for the NSOT patients (*P* = 0.562). The majority of SOT and NSOT patients were White (51.4% vs. 49.5%, *P* = 0.799, respectively), men (60.0% vs. 64.8%, *P* = 0.566, respectively), non-smokers (55.7% vs. 48.6%, *P* = 0.581, respectively), with an ASA class III (71.4% vs. 53.3%, *P* = 0.051, respectively). The mean BMI was 29.2 kg/m^2^ (range, 18–51) for the SOT patients and 29.3 kg/m^2^ (range, 17 to 52) for the NSOT patients (*P* = 0.907). The mean CCI was 6.7 (range, 0–18) for the SOT patients and 6.7 (range, 0–20) for the NSOT patients (*P* = 0.999). Of the 70 SOT patients, 34 (48.6%) underwent TKA and 36 (51.4%) underwent THA, whereas among the 210 NSOT patients, 96 (45.7%) underwent TKA and 114 (54.3%) underwent THA. Osteoarthritis was the primary indication in both the SOT (88.6%) and NSOT (91.4%) patient groups. Osteonecrosis of the femoral head was less common, occurring in five SOT patients (7.1%) and 12 NSOT patients (5.7%). Femoral neck fractures were present in three SOT patients (4.3%) and six NSOT patients (2.9%). The distribution of the procedure and procedure indications did not differ between the patients (*P* = 0.782 and 0.757, respectively) (Table 1).

**Table 1:** Baseline patient demographics and clinical characteristics of SOT and matched NSOT patients

Demographic variables	SOT (*n* = 70)	NSOT (*n* = 210)	*P*-value
Mean age [SD] (range), years	63.3 [10.5] (34–83)	64.2 [13.1] (24–91)	0.562
Race, *n* (%) White Black Asian Other/Unknown	36 (51.4) 12 (17.1) 3 (4.3) 19 (27.1)	104 (49.5) 30 (14.3) 14 (6.7) 62 (29.5)	0.799
Smoking status, *n* (%) Current Former Never	4 (5.7) 27 (38.6) 39 (55.7)	13 (6.2) 95 (45.2) 102 (48.6)	0.581
Sex, *n* (%) Female Male	28 (40.0) 42 (60.0)	74 (35.2) 136 (64.8)	0.566
ASA, *n* (%) 1 2 3 4	0 (0.0) 10 (14.3) 50 (71.4) 10 (14.3)	7 (3.3) 82 (39.0) 112 (53.3) 9 (4.3)	0.051
Mean BMI [SD] (range), kg/m^2^	29.2 [6.3] (18–51)	29.3 [5.8] (17–52)	0.907
Mean CCI [SD] (range)	6.7 [3.4] (0–18)	6.7 [4.7] (0–20)	0.999
Procedure, *n* (%) Total knee arthroplasty Total hip arthroplasty	34 (48.6) 36 (51.4)	96 (45.7) 114 (54.3)	0.782
Procedure indication, *n* (%) Osteoarthritis Osteonecrosis of femoral head Femoral neck fracture	62 (88.6) 5 (7.1) 3 (4.3)	192 (91.4) 12 (5.7) 6 (2.9)	0.757

SOT, solid organ transplant patient; NSOT, non-solid organ transplant patients; SD, standard deviation; *n*, Number; %, percentage; CCI, Charlson comorbidity index; BMI, body-mass index; ASA, American society of anesthesiologists

*, Statistical significance, *P* < 0.05

### Characteristics of SOTs

Table 2 summarizes the types of solid organ transplants included in our cohort. In the SOT patient group (*n* = 70), kidney transplants were the most prevalent, accounting for 61.4% (*n* = 43) of cases, with a mean time from transplant to surgery of 6.4 years (range, 0.3–27.6). Among kidney transplant recipients, 24.3% (*n* = 17) received deceased donor kidneys, and 30.0% (*n* = 21) received living donor kidneys. Liver transplants comprised 24.3% (*n* = 17) of the cohort, with a mean time of 10.2 (range, 1–32) years. Heart transplants represented 8.6% (*n* = 6) of cases, with a mean time of 8 years (range, 1.5–21.6). Less common transplant types included lung (4.3%, *n* = 3) and pancreas + kidney (1.4%, *n* = 1) (Table 2).

**Table 2:** Characteristics of SOTs in the study cohort

Transplant type	Number (%) [Total = 70]	Mean time from transplant to surgery, [SD] (range), years
Heart Kidney Deceased donor Living donor Unavailable/unknown Liver Lung Pancreas + Kidney	6 (8.6) 43 (61.4) 17 (24.3) 21 (30.0) 5 (7.1) 17 (24.3) 3 (4.3) 1 (1.4)	8.0 [8.1] (1.5–21.6) 6.4 [5.8] (0.3–27.6) 5.3 [3.7] (1.2–12.9) 5.1 [4.8] (0.3–20.4) 10.5 [6.0] (4.1–22.1) 10.2 [8.0] (1.0–32.0) 2.1 [0.6] (1.3–2.6) 6.0 [n/a] (n/a)

SOT, solid organ transplant patient; NSOT, non-solid organ transplant patients; n, number; %, percentage; SD, standard deviation; n/a, not available

## Results

### Clinical outcomes and complications

Table 3 presents the differences in clinical outcomes and complication rates between SOT and NSOT patients. The mean duration of clinical follow-up for the study cohort was 2.3 years. The mean duration of surgery was 105 min (range, 39–244) for the SOT patients and 106 min (range, 30–224) for the NSOT patients, comparable between the patient groups (*P* = 0.835). SOT patients experienced significantly longer hospital stays (*P* < 0.001), with a mean duration of 92 h (range, 9–406) compared to 51 h (range, 7–260) for NSOT patients. The effect size of this finding was large (Cohen’s *d* = 0.82). SOT patients were discharged significantly more frequently to skilled nursing facilities (SNF) compared to NSOT patients (15.7% vs. 5.7%, *P* = 0.014). The effect size of this observation was small (Cramer’s *V* = 0.17).

**Table 3:** Comparison of perioperative outcomes and complications between SOT and NSOT

Outcome	SOT (*n* = 70)	NSOT (*n* = 210)	*P*-value (ES)
Mean duration of surgery [SD] (range), minutes	105 [36] (39–244)	106 [30] (30–224)	0.835
Mean length of stay [SD] (range), hours	92 [69] (9-406)	51 [42] (7-260)	<0.001* (0.82)
Discharge disposition, *n* (%) Home Skilled nursing facility Acute rehab facility	56 (80.0) 11 (15.7) 3 (4.3)	194 (92.4) 12 (5.7) 4 (1.9)	0.014* (0.17)
90 day readmissions, *n* (%) Surgical Acute posthemorrhagic anemia PJI Dislocation Non-surgical Abdominal pain/diverticulitis Cerebrovascular accident Chest pain Gout flare Lower back pain Respiratory failure Sepsis (no evidence of PJI) Shortness of breath Cellulitis Urinary tract infection Pain in contralateral limb	11 (15.7) 1 (1.4) 1 (1.4) 0 (0.0) 0 (0.0) 10 (14.3) 1 (1.4) 1 (1.4) 1 (1.4) 1 (1.4) 1 (1.4) 1 (1.4) 2 (2.8) 1 (1.4) 0 (0.0) 1 (1.4) 0 (0.0)	14 (6.7) 5 (2.4) 0 (0.0) 4 (1.9) 1 (0.5) 9 (4.3) 2 (1.0) 1 (0.5) 0 (0.0) 0 (0.0) 0 (0.0) 0 (0.0) 1 (0.5) 1 (0.5) 2 (1.0) 1 (0.5) 1 (1.0)	0.040* (0.12) 0.999 0.010* (0.16)
All-cause revision, *n* (%) Aseptic Dislocation (THA only) Instability (TKA only) Aseptic loosening (THA only) Septic DAIR Two-stage revision arthroplasty	3 (4.3) 2 (2.9) 1 of 36 (2.8) 1 of 34 (2.9) 0 (0.0) 1 (1.4) 0 (0.0) 1 (1.4)	8 (3.8) 4 (1.9) 1 of 114 (0.9) 1 of 96 (1.0) 2 of 114 (1.8) 4 (1.9) 4 (1.9) 0 (0.0)	0.999 0.642 0.999

SOT, solid organ transplant patient; NSOT, non-solid organ transplant patients; n, number; %, percentage; SD, standard deviation; PJI, periprosthetic joint infection; DAIR, debridement, antibiotics, irrigation and implant retention; *, statistical significance, *P* < 0.05; ES, effect size calculated for Cohen’s *d* (numerous parameters: 0.2–0.5 small, 0.5–0.8 medium, > 0.8 large) and Cramer’s *V* (categorical parameters: 0.1–0.3 small, 0.3–0.5 medium, > 0.5 large)

Ninety-day all-cause readmission rates were significantly higher in the SOT patient group compared to the NSOT patients (15.7% vs. 6.7%, *P* = 0.040). The effect size of this finding was small (Cramer’s *V* = 0.12). This elevated readmission rate in SOT patients was primarily driven by non-surgical reasons (14.3% vs. 4.3%, *P* = 0.010, Cramer’s *V* = 0.16), whereas surgical readmissions were similar between SOT and NSOT patients (1.4% vs. 2.4%, *P* = 0.999).

All-cause revision rates of SOT patients were comparable to NSOT patients (4.3% vs. 3.8%, *P* = 0.999), including aseptic (2.9% vs. 1.9%, *P* = 0.642) and septic (1.4% vs. 1.9%, *P* = 0.999) causes (Table 3).

## Discussion

In SOT recipients, TJA can significantly improve quality of life and physical function, which may otherwise be impaired by years of systemic illness and reduced activity before and after transplantation [18]. Our study demonstrated that while SOT patients undergoing primary TJA experience significantly longer hospital stays and higher 90 day all-cause readmission rates, primarily for non-surgical reasons, their all-cause revision rates (both aseptic and septic) are comparable to those of propensity-matched NSOT patients. This suggests that despite increased short-term medical, non-surgery-related complexities, TJA remains a viable and safe procedure for this medically complex population.

SOT recipients are recognized to be at elevated risk for postoperative complications following TJA, owing to their complex medical profiles, chronic immunosuppressive therapy, and cumulative burden of comorbidities [16, 19, 20]. Immunosuppressive agents, while essential to prevent graft rejection, compromise the host immune response and impair tissue healing, thereby increasing susceptibility to PJI and other postoperative complications [16, 21]. Moreover, these agents can exacerbate or induce systemic conditions such as osteoporosis, renal dysfunction, and diabetes mellitus, further complicating perioperative risk stratification and management [22, 23]. Several studies compared outcomes across specific types of SOTs. While Cavanaugh et al. [24] demonstrated a higher risk for postoperative complications in kidney recipients compared to non-kidney recipients, Kuo et al. [25] and Douglas et al. [26] found no difference in complication rate between liver transplant and non-liver transplant patients. Lung, heart, and pancreas transplants were found to have an increased risk of complications compared to NSOT patients [24]. Studies examining outcomes by specific organ type are relatively limited and show considerable variability. While Duplantier et al. [27] found that kidney recipients had significantly higher complication rates compared to liver recipients, a large database study by Klement et al. [28] reported that renal transplant patients undergoing THA had the highest risk of complications, while lung recipients had outcomes comparable to NSOT patients. In TKA literature [17], transplant patients overall had increased 90 day complications, PJI, periprosthetic fractures, and revision rates, with heart and lung recipients experiencing better outcomes than kidney and pancreas recipients. Taken together, these findings underscore the need for individualized perioperative planning to address the distinct risks associated with each type of organ transplant. These elevated risks in SOT patients often translate into longer hospital stays, more intensive monitoring, and the need for multidisciplinary coordination, particularly in the early postoperative period. Accordingly, comprehensive preoperative evaluation—including optimization of metabolic and cardiovascular status, assessment of graft function, and adjustment of immunosuppressive regimens—is crucial. In addition, tailored perioperative protocols, such as individualized antibiotic prophylaxis and enhanced vigilance for atypical presentations of infection, are paramount in mitigating the elevated risk of adverse events in this population [30, 31]. Despite these challenges, with careful patient selection and optimized perioperative pathways, favorable surgical outcomes remain achievable in this vulnerable cohort.

Beyond the known immunologic and systemic vulnerabilities associated with solid organ transplantation, our findings underscore important clinical differences in healthcare utilization and postoperative outcomes between SOT and NSOT patients undergoing TJA. In this study, SOT patients exhibited a significantly longer mean hospital stay compared to their propensity-matched NSOT counterparts (92 vs. 51 h, *P* < 0.001). This extended length of stay likely reflects the need for more intensive postoperative monitoring and management of their complex medical conditions, which can influence healthcare costs and resource utilization [19]. This finding is consistent with other studies [15, 19, 32] that reported longer hospital stays for SOT patients undergoing TJA. Despite previous reports, findings of our study demonstrate a rather greater difference in length of stay between SOT and NSOT patients (+ 41 h vs. 15.3 [19] vs. 12 [33] hours). Furthermore, the 90 day all-cause readmission rates were markedly higher in the SOT group (15.7% vs. 6.7%, *P* = 0.040), predominantly driven by non-surgical reasons (14.3% vs. 4.3%, *P* = 0.010). This finding highlights the vulnerability of SOT patients to systemic medical complications such as acute posthemorrhagic anemia, aspiration pneumonia, and sepsis, rather than surgical site issues. Other large database studies [21, 34] have similarly reported increased readmission rates in SOT patients following TJA, often highlighting medical comorbidities as primary drivers. A large Nationwide Inpatient Sample analysis by Navale et al. [19] reported an increased complication risk (odds ratio 1.56), longer length of stay, and increased admission costs in SOT patients compared to NSOT patients undergoing THA. Nonetheless, despite the higher rate of 90 day non-surgical related readmissions, our study found that SOT patients’ rate of all-cause revisions (4.3% vs. 3.8%, *P* = 0.999), including both aseptic (2.9% vs. 1.9%, *P* = 0.642) and septic revisions (1.4% vs. 1.9%, *P* = 0.999), was comparable to NSOT patients. While studies by Klement et al. [17] and Labaran et al. [35] have indicated a higher risk of revision, particularly for PJI, in SOT patients after primary and revision TJA, our findings align with others who suggest similar revision rates when accounting for patient comorbidities through rigorous matching. The strength of this study lies in its comparison of propensity-matched groups based on patient demographics, particularly CCI, which accurately reflects the patients’ actual health status. To our knowledge, this rigorous methodology has not been extensively performed in institutional data for total hip and knee arthroplasty. This highlights that TJA is a safe procedure in SOT patients, demonstrating a very low revision rate over a mean follow-up time of 2.3 years, especially concerning revision surgery.

### Limitations

This study is subject to several limitations. As a retrospective analysis, it relies on the accuracy and completeness of electronic medical records, with the potential for unrecognized data omissions, especially regarding complications managed at outside institutions. Although we employed robust PSM to control for key demographic and comorbidity variables, unmeasured confounders may still have influenced our findings. Importantly, like prior studies, we were unable to assess the specific immunosuppressive regimens used by SOT patients before, during, or after surgery. Variations in immunosuppressive class, dosage, and duration may have contributed to complications such as infection or delayed healing but were not consistently available for inclusion. Additionally, clinical data on graft function, transplant rejection history, or perioperative immunologic status were not collected, despite their potential relevance. The relatively small sample size of the SOT cohort limited statistical power to detect differences in low-frequency outcomes such as septic revision, and the mean follow-up of over two years, while adequate for early and midterm outcomes, does not capture long-term survivorship. Furthermore, the findings may have limited generalizability, as all procedures were performed at tertiary referral centers by fellowship-trained arthroplasty surgeons within multidisciplinary teams that included transplant, infectious disease, and internal medicine specialists. Outcomes in other practice settings may therefore differ. Nonetheless, this study has several notable strengths. It represents one of the largest single-institution analyses to date of TJA outcomes in SOT patients, with comprehensive chart-level data review and a well-defined cohort of kidney, liver, and heart transplant recipients. Additionally, detailed documentation of readmissions, including a breakdown of surgical vs. non-surgical causes, enabled a nuanced understanding of the unique postoperative challenges faced by SOT recipients. These methodological strengths support the generalizability of our findings and reinforce the conclusion that, when managed in a structured multidisciplinary setting, TJA is a safe and effective option for appropriately selected solid organ transplant recipients.

## Conclusions

In this propensity-matched analysis of patients undergoing TJA, SOT patients demonstrated comparable rates of surgical complications and revisions to non-transplant patients, despite a significantly higher rate of 90 day readmissions primarily due to non-surgical causes. These findings suggest that, with appropriate perioperative planning and multidisciplinary care, TJA can be performed safely in SOT recipients with acceptable early outcomes. The low incidence of septic and aseptic revisions over a mean follow-up of 2.3 years supports the role of arthroplasty as a viable and durable intervention in this medically complex population. Continued efforts to optimize medical management and reduce readmission risk will further improve outcomes in this growing patient group.

## Data Availability

No datasets were generated or analysed during the current study.
